# Combined treatment of a giant duodenal gastrointestinal stromal tumor: A case report

**DOI:** 10.1097/MD.0000000000043522

**Published:** 2025-07-25

**Authors:** Qian Feng, Jianxiong Lai

**Affiliations:** a Department of Hepatobiliary Surgery, Deyang People’s Hospital, Deyang, Sichuan Province, China; b Department of General Surgery, The Third Hospital of Mianyang (Sichuan Mental Health Center), Mianyang, Sichuan Province, China.

**Keywords:** duodenal gastrointestinal stromal tumor, multidisciplinary team, neoadjuvant therapy, operation, targeted therapy

## Abstract

**Rationale:**

Gastrointestinal stromal tumors (GISTs) in the duodenum are usually very rare, accounting for only 3% to 5% of all GISTs. Giant duodenal GISTs with a more than 10 cm diameter are even rarer, and there is currently no consensus on their treatment. This article reports a case of successful treatment of a giant duodenal GIST.

**Patient concerns:**

A 59-year-old woman presented with mid-upper abdominal pain accompanied by nausea and bloating. We underwent contrast-enhanced abdominal computed tomography scans and computed tomography-guided fine needle aspiration biopsy.

**Diagnoses:**

The final diagnosis was duodenal GIST with a risk stratification of high-risk.

**Interventions:**

After a multidisciplinary team discussion, the patient underwent a combination of targeted-surgical-targeted therapy.

**Outcomes:**

The patient ultimately achieved R0 resection at surgery, and no recurrence was noted during the follow-up period.

**Lessons:**

Targeted-surgery-targeted combined therapy is one of the effective means for giant duodenal GIST. At the same time, genetic testing before neoadjuvant therapy is crucial for guiding treatment and is strongly recommended as a basic examination method.

## 1. Introduction

Gastrointestinal stromal tumors (GISTs) are a type of tumor that originates from Cajal cells or their precursors in the gastrointestinal muscularis and occur most frequently in the stomach (50–60%), whereas duodenal GISTs account for only 3% to 5% of cases, while giant lesions (>10 cm in diameter) are extremely rare.^[1–3]^ This rarity creates a knowledge gap on the best management strategies. In this case, we share our experience in the combined treatment of a giant duodenal GIST.

## 2. Case presentation

A 59-year-old woman presented to the hospital with a 1-week history of mid-upper abdominal pain accompanied by nausea and bloating. The patient underwent a total hysterectomy 15 years before admission and recovered well after surgery. She has never smoked or drank alcohol and does not have any family history of genetic disorders. Vital signs on admission were as follows: blood pressure, 129/87 mm Hg; heart rate, 78 beats/min; respiratory rate, 20/min; body temperature, 37.0°C; and body mass index, 32.46 kg/m². The physical examination suggested light tenderness in the upper abdomen without significant rebound tenderness, as well as muscle tension. At the same time, a visual examination showed that the patient had abundant abdominal fat. The rest of the physical examination showed no abnormalities. Laboratory tests showed that the patient’s hemoglobin level on admission was 76 g/L (reference range: 115–150 g/L for women), platelet count was 423 × 10^9^/L (reference range: 100–300 × 10^9^/L for women), low-density lipoprotein cholesterol was 3.86 mmol/L (reference range: <3.37 for women), and high-density lipoprotein cholesterol was 0.76 mmol/L (reference range: >1.04 for women). Other routine hematological and biochemical profiles, such as tumor markers, liver function, and kidney function, were within normal limits.

**Figure 1.**
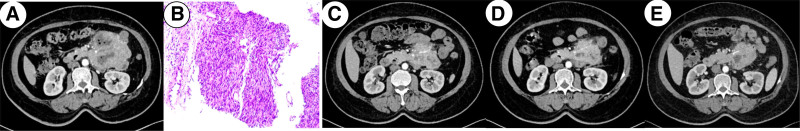
Preoperative examination data of this patient. (A) Contrast-enhanced CT image of the abdomen obtained when the patient was first admitted to this hospital (arterial phase). (B) Pathological image of CT-guided fine needle aspiration biopsy (hematoxylin and eosin stain ×100 magnification). (C) Contrast-enhanced CT image of the abdomen obtained during the first follow-up of the patient during neoadjuvant therapy. (D) Contrast-enhanced CT images of the abdomen obtained during the second follow-up of the patient during neoadjuvant therapy. (E) Contrast-enhanced CT images of the abdomen obtained during the third follow-up of the patient during neoadjuvant therapy. CT = computed tomography.

**Figure 2.**
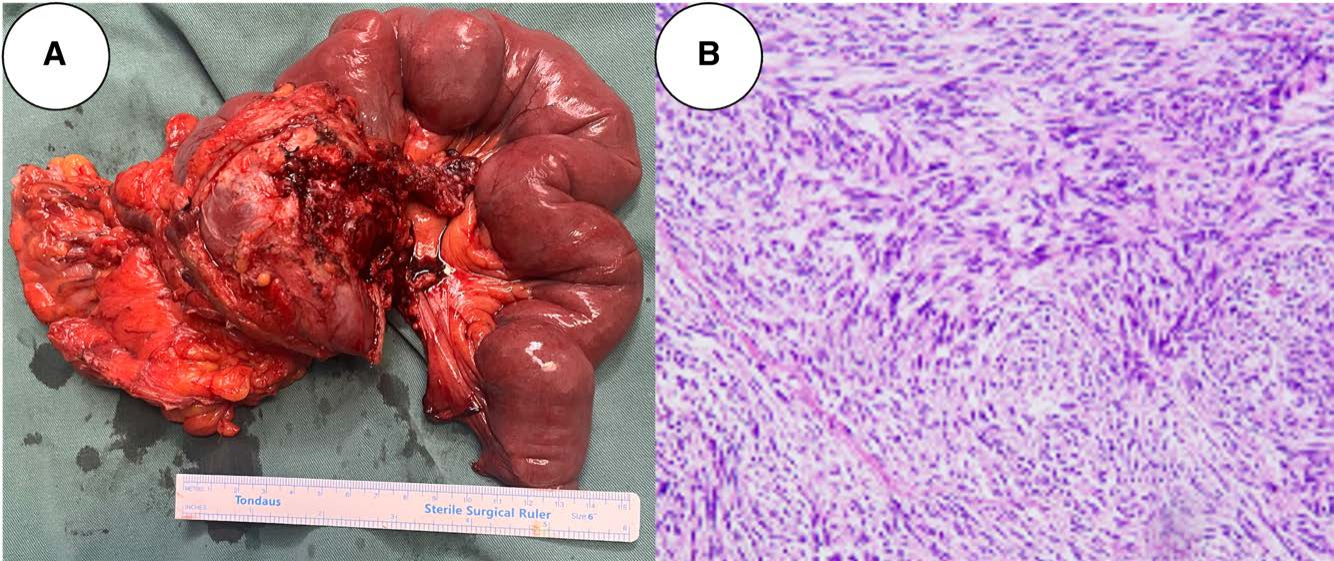
Postoperative examination data of this patient. (A) Specimens removed during surgery. (B) Pathological images of surgically resected specimens (hematoxylin and eosin stain ×100 magnification).

Contrast-enhanced computed tomography (CT) scan of the abdomen suggested a space-occupying lesion in the left epigastrium, poorly demarcated from the horizontal and ascending segments of the duodenum, measuring approximately 10.2 cm × 8.1 cm × 8.4 cm (Fig. 1A), and no suspicious lymph node metastases were detected. To further clarify the diagnosis, we performed a CT-guided fine needle aspiration biopsy. The pathological examination results suggested a spindle cell tumor (Fig. 2B). Further immunohistochemical examination showed that it was CD117 positive, discovered on gist-1 positive, smooth muscle actin positive, and CD34 positive. The final diagnosis was a stromal tumor.

Based on the above information, we organized a multidisciplinary team discussion. First, the radiologist read the patient’s abdominal contrast-enhanced CT images again and concluded that the tumor was located in the left upper abdomen and had no obvious boundary with the ascending and horizontal segments of the duodenum, so it was considered that the tumor was likely to originate from the duodenum. At the same time, because the tumor was huge and closely related to the pancreas, the possibility of pancreatic invasion could not be ruled out. Second, the gastrointestinal surgeon believed that the patient’s duodenal GIST was large and had no obvious boundary with the pancreas. It would be difficult to achieve R0 resection during surgery, and the pancreas might even need to be removed at the same time. The surgery was very traumatic, so the patient was recommended to undergo neoadjuvant therapy. Third, the pathologist believed that the pathological results suggested a spindle cell tumor, and immunohistochemistry showed that CD117 was positive, discovered on gist-1 was positive, smooth muscle actin was positive, and CD34 was positive. However, since the tissue sent for examination was a puncture specimen, risk stratification could not be performed. The current diagnosis was a giant duodenal GIST, and the first-stage surgery could not achieve R0 resection. Neoadjuvant therapy was recommended. At the same time, before treatment, the patient was advised to complete genetic testing, especially the detection of c-KIT proto-oncogene protein and platelet-derived growth factor receptors (PDGFRA) gene mutations. These 2 genes are very meaningful in guiding medication. Finally, the oncologist expressed his opinion: the diameter of the patient’s duodenal stromal tumor was more than 10 cm. According to the relevant guidelines and the principle of organ function protection, neoadjuvant treatment was recommended for the patient. Before that, genetic testing was recommended. If there were no special circumstances, the drug and dosage could be imatinib 400 mg/d. The treatment time was controlled at 6 to 12 months, and surgery was performed in time according to the tumor regression. We then expressed the results of the multidisciplinary team’s discussion to the patient. Considering economic factors, the patient refused genetic testing but agreed to the neoadjuvant treatment plan. Finally, we started the neoadjuvant targeted therapy of imatinib 400 mg/d for the patient.

The patient underwent a follow-up examination 2 months after oral drug therapy, and we performed a contrast-enhanced CT scan of the abdomen. The results of this examination suggested that there was a significant shrinkage of the tumor, with a tumor size of 6.4 cm × 6.1 cm × 5.6 cm (Fig. 1C). Therefore, we recommended that the patient continue to follow the original regimen to continue treatment. Two months later, the patient underwent a second review. Unfortunately, the results of this review showed that the tumor size had not changed significantly compared with the previous one, and the tumor size was about 6.2 cm × 6 cm × 5.4 cm (Fig. 1D). We recommended that the patient increase the drug dose to 600 mg/d. However, over the next 2 weeks, the patient experienced adverse drug reactions, primarily in the form of loss of appetite, nausea, and bilateral ankle edema. We suppressed nausea by oral ondansetron and gave the patient measures such as low-dose furosemide (20 mg/d) and restriction of natriuretic salt intake, and the patient’s symptoms resolved, so we evaluated and continued to give imatinib orally and did not adjust the drug dose. After 3 months of continuous treatment with 600 mg/d of imatinib, the patient underwent a third follow-up examination, and this time, the results suggested that the tumor size was still unchanged (tumor size: 6.2 cm × 6 cm × 5.4 cm; Fig. 1E). We realized that the tumor had developed drug resistance and that continuing neoadjuvant therapy might not be effective, so we chose to terminate neoadjuvant therapy and prepare for surgical treatment. According to the Choi evaluation criteria,^[4]^ partial remission was defined as: A decrease in size of ≥10% or a decrease in tumor density (HU) ≥15% on CT. No new lesions. No obvious progression of nonmeasurable disease. In this case, the maximum diameter decreased from 10.2 to 6.2 cm (39% decrease), thus meeting the criteria for partial remission.

Before the operation, we invited the radiologist to read the patient’s latest contrast-enhanced CT results. The results showed that the tumor was shrunk, and the boundary with the pancreas was clear. Considering that the tumor was located in the third and fourth parts of the duodenum, we finally performed a segmental duodenectomy with end-to-side duodenojejunostomy. The specimen after surgery is shown in Figure 2A. Histopathological examination showed a final tumor size of 7.8 cm × 6 cm × 5.5 cm with mitotic count <3/50 high power field and negative margins. The final risk classification was high-risk (Fig. 2B). The operation went smoothly. The patient was hospitalized for 13 days after the operation without any postoperative complications. Given the high-risk nature of this tumor, we believe that long-term surveillance is very necessary, and therefore, we give the following recommendations for this patient: Continue oral imatinib 400 mg/d for 3 years after surgery. Abdominal/pelvic CT with contrast every 3 months for 3 years and then annually. Annual chest CT to exclude pulmonary metastases. Clinical assessment (symptom review, physical exam) at each imaging interval. Consideration of PET-CT if recurrence is suspected.

Up to now, the follow-up has lasted for about 18 months, and no tumor recurrence has been found. The patient’s course of treatment is summarized in Table 1. For the publication of this case, we have obtained informed consent from the patient. As the article did not disclose any sensitive personal information about the patient, mainly reviewed the patient’s diagnosis and treatment, and did not cause any harm to the patient, the hospital’s ethical committee concluded that the manuscript did not require ethical review.

**Table 1:** Summary of the patient’s treatment process.

Time point	Clinical phase	Key events or interventions	Outcomes
Day 0	Initial presentation	Abdominal pain, nausea, and bloating	CT: 10.2 cm duodenal mass; CT-guided FNA → GIST
Week 1	Diagnosis	CD117+/DOG-1+/SMA+/CD34+	High-risk GIST
Month 1–2	Phase I neoadjuvant therapy	Imatinib 400 mg/d	Tumor size (10.2 cm → 6.4 cm)
Month 3–4	Phase II neoadjuvant therapy	Imatinib 400 mg/d	Tumor size (6.4 cm → 6.2 cm)
Month 5–7	Phase III neoadjuvant therapy	Imatinib 600 mg/d	Tumor size (6.2 cm → 6.2 cm); partial remission (choi evaluation criteria)
Month 7	Surgery	Segmental duodenectomy	R0 resection
Pathology	–	Final size: 7.8 cm; free margins; mitotic count: <3/50 HPF	Adjuvant imatinib indicated
Month 8–26	Adjuvant therapy	Imatinib 400 mg/d (36 months)	Monitoring every 3 mo
Month 26	Follow-up	CT: no recurrence	Continue to follow-up
Beyond month 26	Long-term surveillance	CT/clinical monitoring	–

CT = computed tomography, CD = cluster of differentiation, DOG = discovered on gist, FNA = fine needle aspiration, GIST = gastrointestinal stromal tumors, HPF = high power field, SMA = smooth muscle actin.

## 3. Discussion

This case describes in detail the course of a patient with a giant mesenchymal tumor of the duodenum from its onset to the end of treatment. Unlike small asymptomatic duodenal GISTs,^[5]^ large tumors usually cause gastrointestinal bleeding or epigastric discomfort.^[6]^ However, this patient, even though the tumor was huge, still had only mild pain in the upper and middle abdomen with no warning symptoms, thus delaying medical attention. Although CT may occasionally mislocate tumors,^[7]^ it remains the gold standard for the National Comprehensive Cancer Network/ European Society Of Medical Oncology guidelines,^[8,9]^ and as our case demonstrates, guides successful biopsy and response assessment. Tumor size and location are often important factors that influence treatment options for GIST. For large tumors, surgery may require the difficult choice of removing other organs or tumor remnants. Therefore, neoadjuvant therapy has become a new option for the treatment of giant GIST because of its potential to increase the complete resection rate of the tumor and, more importantly, reduce the tumor rupture rate. Imatinib is one of the most commonly used drugs in the neoadjuvant therapy of GIST, and the usual initial dose is 400 mg/d. Higher doses of imatinib, such as 600 mg/d or 800 mg/d, have also been studied in the early stages of neoadjuvant therapy, but the results were not significantly better than 400 mg/d and instead increased many of the associated complications.^[8]^ Therefore, for giant GIST, we recommend neoadjuvant therapy for patients, and the drug and dose are imatinib 400 mg/d. In the early stage of drug use, the patient’s tumor shrank significantly, but as time went by, the patient’s tumor diameter tended to stabilize. Even if we adjusted the drug dose to 600 mg/d, it only increased the patient’s related complications and did not have a significant effect on the tumor. Ultimately, we assessed a partial remission according to the Choi evaluation criteria and performed surgery 2 weeks after discontinuing imatinib.^[4]^ Duodenal GIST is difficult to treat clinically because of its complex anatomy and surgical trauma. The current consensus is that the surgical principle for GIST is complete tumor resection plus clear surgical margins.^[10]^ Because lymph node metastasis is rare in mesenchymal tumors, routine lymph node dissection is not recommended.^[10]^ To date, there is no consensus on the surgical approach to duodenal GISTs. Pancreaticoduodenectomy for duodenal GIST is the choice of most surgeons.^[11]^ However, wedge resection with a 1-stage suture can also be an alternative surgical procedure provided that adequate lumen is preserved.^[12]^ Segmental duodenectomy with end-to-end or end-to-side duodenojejunostomy may also be used for larger tumors in the third and fourth portions of the duodenum.^[13]^ At the same time, partial duodenectomy with Roux-en-Y duodenojejunostomy is also a feasible option.^[14]^ In our case, a segmental duodenectomy combined with end-to-side duodenojejunostomy was performed as in previous cases; however, unlike previous cases, we adopted a neoadjuvant treatment strategy with preoperative use of targeted drugs to downgrade and downstage the tumor and reduce its size, which significantly reduced the difficulty of the surgery and increased the complete resection rate, meanwhile, avoided resection of the pancreas, and maximally preserved organ function.

This patient was successfully treated with neoadjuvant imatinib to shrink the tumor and achieve R0 resection, highlighting the value of targeted therapies in organ function preservation. Although the National Comprehensive Cancer Network guidelines recommend 6 to 12 months of preoperative imatinib treatment, this case reached maximum remission at 7 months,^[15]^ suggesting that the duration of treatment needs to be individualized. At the same time, this patient with giant duodenal GIST avoided pancreaticoduodenectomy and had no recurrence after 18 months of follow-up, which is in line with the concept of “preservation of function” proposed by the European Society Of Medical Oncology guidelines and provides an important reference for the treatment of similar patients.^[16]^

Gene mutation testing is widely used in GISTs due to its importance in predicting the efficacy of targeted drugs and guiding clinical treatment. For example, the PDGFRA exon 18 D842V mutation may lead to primary imatinib resistance, and such patients should be treated early with the second-line drug sunitinib.^[17]^ KIT exon 13, 14, 17, and 18 mutations may lead to secondary resistance and thus affect the subsequent efficacy of imatinib.^[18]^ Patients with KIT exon 9 mutations benefited more from the higher 800 mg/d imatinib dose.^[17]^ Unfortunately, due to funding constraints, our cases lacked c-KIT/PDGFRA mutation analysis, which is a shortcoming of this case. We could not check whether the patient had KIT exon 9 mutation and could only use 400 mg/d of imatinib based on experience. Although the tumor shrank significantly in the early stage of neoadjuvant therapy, it was in a stable state after several months of treatment. At this time, we could not determine whether there was secondary drug resistance. Therefore, we could not rashly use the second-line drug sunitinib to continue neoadjuvant therapy. Of course, if a change in condition occurs in the future, we will strongly recommend that the patient complete genetic testing at that time.

## 4. Conclusion

Abdominal masses of unknown nature need to include GIST as one of the differential diagnoses. Targeted-surgical-targeted combination treatment modality is one of the effective means for giant duodenal GIST, however, for neoadjuvant treatment of giant GIST, genetic testing is strongly recommended as a routine pretreatment screening tool for precision treatment.

## Author contributions

**Investigation:** Qian Feng.

**Supervision:** Jianxiong Lai.

**Writing – original draft:** Qian Feng.

**Writing – review & editing:** Jianxiong Lai.
